# Selected Post-Translational Modifications—Phosphorylation and Glutathionylation—As Factors Involved in the Regulation During the Pregnancy Course and Foetal Membrane Release in Cows

**DOI:** 10.3390/ijms262210984

**Published:** 2025-11-13

**Authors:** Jacek Wawrzykowski, Monika A. Jamioł, Ewelina Kosztowny, Marta Kankofer

**Affiliations:** Department of Biochemistry, Faculty of Veterinary Medicine, University of Life Sciences in Lublin, Akademicka 12, 20-033 Lublin, Poland; jacek.wawrzykowski@up.lublin.pl (J.W.); monika.jamiol@up.lublin.pl (M.A.J.); ewelina.kosztowny@up.lublin.pl (E.K.)

**Keywords:** bovine placenta, phosphorylation and glutathionylation post-translational modifications, retention of foetal membranes

## Abstract

Post-translational modifications (phosphorylation and glutathionylation) not only assure protein diversity but are also responsible for the controlling of the biological activity of selected proteins in health and disease. The aim of the study was to monitor the profile of changes in molecular weight of proteins undergoing selected post-translational modifications by measurement of the intensity of phosphorylation and glutathionylation within the pregnancy course and parturition in cows with and without the retention of foetal membranes. The intensity of selected post-translational modifications was measured in bovine placental tissues collected during pregnancy (2nd, 4th, 5th, and 7th month, *n* = 4 per month) and parturition (not-retained foetal membranes (NRFM, *n* = 6) and retained foetal membranes (RFM, *n* = 6). Placental tissues were homogenised and used for the Phosphoprotein Phosphate Estimation Assay Kit and Western blotting analyses with adequate antibodies. The content of phosphorylated proteins was significantly higher (*p* < 0.05) in the 2nd month as compared to other months, both in the maternal and foetal parts of the placenta. Moreover, no significant differences were observed between NRFM and RFM samples. The results of Western blotting showed the shift in molecular weight and indirect content of phosphorylated selected amino acids. Further research on the role of post-translational modifications in pregnancy and parturition may give new insight into their biochemical regulation.

## 1. Introduction

Post-translational modifications (PTMs) are crucial regulatory mechanisms that alter protein properties through the covalent attachment of chemical groups to specific amino acid residues [1]. By increasing the chemical variability of proteins, PTMs extend their functional range beyond the limitations of the genetic code [2]. They modulate protein conformation, activity, localization, turnover, stability, and interactions, thereby expanding the diversity of the proteome and regulating numerous cellular processes under both physiological and pathological conditions [1,3]. They depend on the primary structure of proteins. Many PTMs are dynamic and reversible, enabling rapid cellular responses to environmental and developmental signals without requiring protein degradation or de novo synthesis [4]. Although hundreds of PTM types have been described, experimental data are dominated by a few well-studied modifications, mainly phosphorylation [5].

Phosphorylation involves the reversible addition of a phosphate group to serine, threonine or tyrosine residues, but can also target less common residues [2]. The balance between phosphorylation and dephosphorylation, regulated by kinases, phosphatases and the energy state of the cell, underlies the mechanisms controlling processes such as protein synthesis, signal transduction, cell division and ageing. By modulating the activity of enzymes and receptors, phosphorylation allows cells to adapt quickly to changing conditions [6].

Another important but less-explored PTM is protein glutathionylation (GS-ylation), a reversible post-translational modification involving the formation of a disulfide bond between protein cysteine and the cysteine of glutathione (GSH). Both enzymatic and non-enzymatic mechanisms contribute to this process [7]. The reversal of the reaction, called de-glutathionylation, is possible thanks to the activity of thiol-disulfide oxidoreductases or redoxins [8]. Increased GS-ylation commonly reflects heightened oxidative stress, resulting in damage to proteins, DNA, and lipids [9]. The biological role of GS-ylation involves modulation of the structure and function of target proteins, primarily enzymes, typically leading to their inhibition, although in some cases it may enhance their activity [8]. As a redox signalling mechanism, GS-ylation interacts with the phosphorylation mechanism, modulating the activity of kinases and phosphatases in the cellular response to oxidative stress [9].

During pregnancy, maternal metabolism undergoes significant adjustments, frequently associated with dynamic remodeling of tissue-specific protein functions and turnover. Phosphorylation-dependent mechanisms have been implicated in the regulation of key reproductive processes in dairy cows, including lactation [10,11], placental steroidogenesis [12], and the function of extracellular matrix (ECM) proteins. For example, osteopontin is phosphorylated and essential for adhesion and signal transduction at the uterine-placental interface [13].

The retention of foetal membranes (RFM) is a risk factor for the development of postpartum uterine infections, including metritis and clinical endometritis [14]. These conditions reflect the complex interplay between immune response, redox state, and protein-level regulation, highlighting the relevance of PTMs in the physiology and pathology of pregnancy.

Therefore, the hypothesis was stated that selected PTMs may be altered in cases of foetal membrane retention and be involved in this process. The study aimed to monitor the profile of changes in molecular weight of proteins undergoing selected PTMs by measurement of the intensity of phosphorylation and glutathionylation during the pregnancy course and parturition in cows with and without the retention of foetal membranes. These PTMs were selected for this study, because protein glutathionylation is frequently highlighted in the literature as functionally analogous to protein phosphorylation in terms of regulatory potential.

## 2. Results

### 2.1. Phosphoprotein Quantification

**Figure 1:**
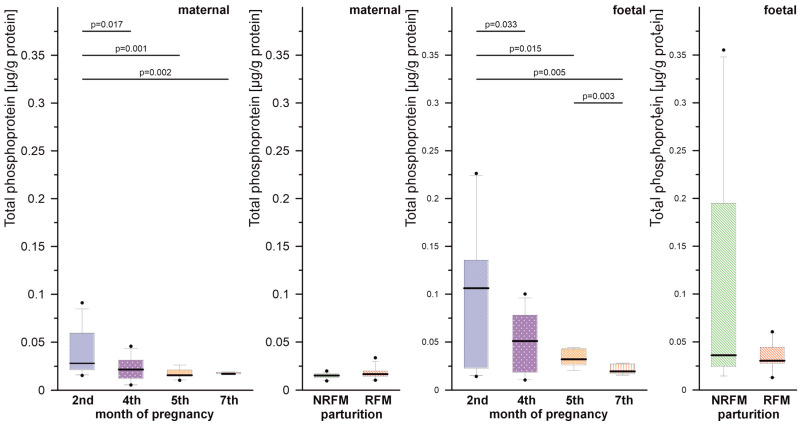
Total phosphoprotein content in cow placenta samples. Only results with statistically significant differences (*p* < 0.05) are marked, outliers are marked with ●.

The results of the measurement of total protein phosphorylation in placental samples during pregnancy and parturition are presented in Figure 1.

In the maternal part, the amount of phosphorylated proteins in the 2nd month of pregnancy was significantly higher (*p* < 0.05) compared to the subsequent months. A similar trend was noted in the foetal part, where protein phosphorylation in the 4th, 5th, and 7th months was significantly lower (*p* < 0.05) than in the 2nd month. Additionally, a significant decrease (*p* < 0.05) in phosphorylated proteins’ level was observed in the 7th month compared to the 5th month in the foetal compartment.

There was a significant negative correlation between gestational age and the amount of phosphorylated proteins in the maternal (Spearman’s ⍴ = −0.454; 95% CI: −0.713 to −0.266; *p* < 0.001) and foetal (Spearman’s ⍴ = −0.392; 95% CI: −0.650 to −0.179; *p* < 0.001) part of the placenta.

No significant differences in the level of phosphorylated proteins were observed between cows with normal placental expulsion and those with retained placenta.

### 2.2. Post-Translational Modifications

Post-translational modifications within proteins in samples from pregnancy and parturition were presented as the results of WB densitometric analysis obtained for the following modifications: proteins with phosphorylated tyrosine, serine and threonine and protein glutathionylation (GS-ylation). Supplementary Materials presents examples of analysis results for selected bovine placental samples: SDS-PAGE (Figure S1), WB (Figures S2–S5) and β-actin loading control (Figure S6). The results were standardized to the protein content per lane.

#### 2.2.1. Phosphorylated Tyrosine

**Figure 2:**
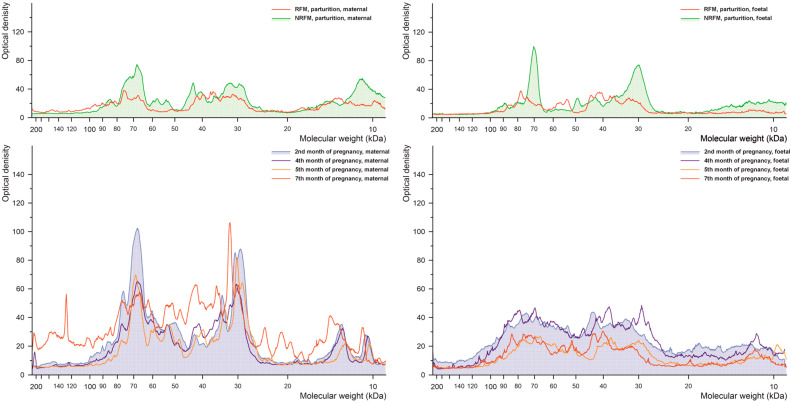
Densitometric analysis of the proteins with phosphorylated tyrosine after SDS-PAGE separation of the bovine placental proteins.

The results of densitometric analysis of cow placenta proteins separated by electrophoresis during pregnancy and parturition for tyrosine phosphorylation are shown in Figure 2. Similar patterns of tyrosine-phosphorylated proteins are observed in the 2nd, 4th and 5th months of pregnancy in both maternal and foetal parts of the placenta, although significant differences in signal intensity between these parts are present. In the 7th month of pregnancy, a distinct shift in molecular weight is observed for proteins of 70 kDa. Significant differences between NRFM and RFM samples are visible for proteins of 70, 55, and 10 kDa in the maternal part and 70, 30 kDa in the foetal part.

#### 2.2.2. Phosphorylated Serine

**Figure 3:**
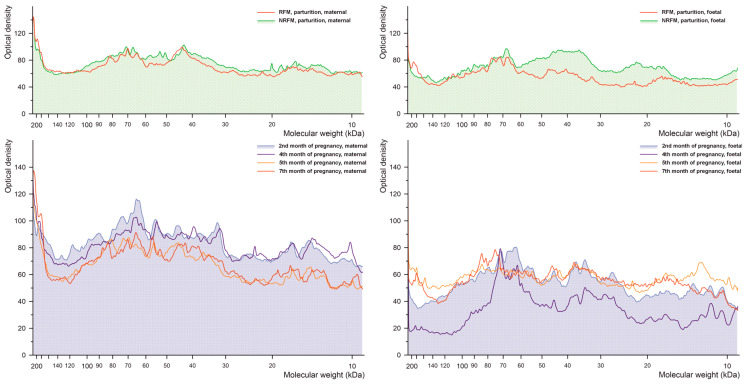
Densitometric analysis of the proteins with phosphorylated serine after SDS-PAGE separation of the bovine placental proteins.

The results of densitometric analysis of cow placenta proteins separated by electrophoresis during pregnancy and parturition for serine phosphorylation are shown in Figure 3. Proteins phosphorylated on serine display a more stable profile than those phosphorylated on tyrosine, although differences between the maternal and foetal parts are evident. A shift in molecular weight associated with phosphorylation-related attachment or removal of phosphate groups is observed in NRFM and RFM samples for proteins of approximately 40 kDa in the maternal part and approximately 75 kDa in the foetal part.

#### 2.2.3. Phosphorylated Threonine

**Figure 4:**
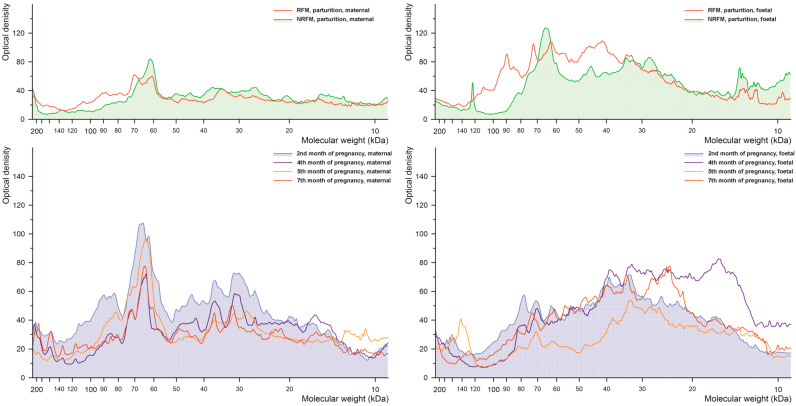
Densitometric analysis of the proteins with phosphorylated threonine after SDS-PAGE separation of the bovine placental proteins.

The results of densitometric analysis of cow placenta proteins separated by electrophoresis during pregnancy and parturition for threonine phosphorylation are shown in Figure 4. The profile of threonine-phosphorylated proteins differs primarily between NRFM and RFM samples, with changes observed in proteins of approximately 60–80 kDa in the maternal part and approximately 20–100 kDa in the foetal part. Profiles between maternal and foetal parts differ significantly.

#### 2.2.4. Glutathionylated Proteins

**Figure 5:**
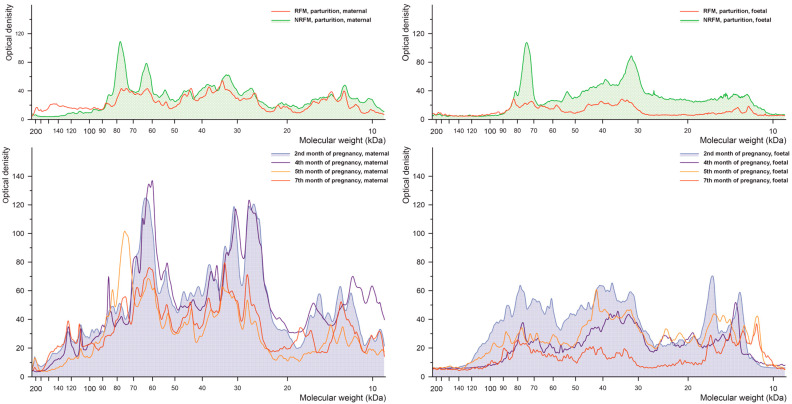
Densitometric analysis of the glutathionylated proteins after SDS-PAGE separation of the bovine placental proteins.

The results of densitometric analysis of cow placenta proteins separated by electrophoresis during pregnancy and parturition for protein glutathionylation (GS-ylation) are shown in Figure 5. The profile of glutathionylated proteins is similar in the 2nd and 4th month of pregnancy in both placental parts. In the 4th and 7th months, clear differences and molecular weight shifts are observed for proteins of 70–80, 20–30 kDa in the maternal part and 30–40, 10–20 kDa in the foetal part. Differences between NRFM and RFM samples are even more pronounced, particularly for proteins of 60–80 kDa in the maternal part and 70–80, 30–40 kDa in the foetal part.

## 3. Discussion

The present study describes the profile of protein phosphorylation and glutathionylation in bovine placenta during pregnancy, as well as physiological and disturbed release of foetal membranes. Our results demonstrate significant and dynamic shifts in protein molecular weights and content across pregnancy and postpartum stages, suggesting that PTMs may change the shape of protein and be associated with the physiological state of the placenta both during gestation and during the release of foetal membranes.

Indeed, PTMs are known to assure the biodiversity of selected proteins, including placental extracellular matrix proteins, thereby influencing their structural and potentially their functional properties modulating their biological activity. Depending on the protein and its amino acid content susceptible to the attachment of additional groups, such modifications may either inhibit or enhance protein function. Any changes in the availability of these amino acids, e.g., tyrosine or cysteine, may modify not only the secondary structure of proteins but also even their biological activity. Such availability can be altered in case of peroxidative damage to selected amino acids caused by oxidative stress [5,15].

One of the most extensively studied PTMs in the placenta is phosphorylation. In dairy cows, reduced neutrophil activity observed in the perinatal period may be related to the modulation of tyrosine phosphorylation of intracellular proteins. Western blot analyses detected the presence of several phosphotyrosine-modified proteins, two of which (42–44 kDa and 90 kDa) showed significant changes in signal intensity depending on the periparturient phase. The 42–44 kDa protein was additionally identified as a phosphorylated form of MAPK kinase, indicating the activation of signalling pathways regulating neutrophil function [16].

However, the specific changes in phosphorylation of ECM proteins in the placenta during different stages of pregnancy remain unclear and require further investigation. Research on phosphorylation in human placentas has shown significant tyrosine phosphorylation throughout gestation, suggesting its importance in placental growth and development [17]. Moreover, the Authors noticed that threonine phosphorylation was predominant in the first trimester, while serine phosphorylation increased toward delivery.

The analysis of the phosphoprotein profile of human placental plasma membrane enabled the identification of protein markers that may be involved in the development of preeclampsia, a serious pregnancy complication [18].

In our study, the analysis of total protein phosphorylation in bovine placental samples revealed a clear decline as pregnancy progressed, both in the maternal and foetal compartments. This suggests that phosphorylation is particularly pronounced during early gestation and may primarily support processes of placental development. However, it should be emphasized that due to the availability of biological material, only selected months of pregnancy were included in the analysis, and therefore the results do not provide a full picture of phosphorylation dynamics throughout the entire pregnancy period. These limitations highlight the need for further studies covering the entire course of pregnancy in cows to fully elucidate the role of phosphorylation in placental function.

Post-translational modifications are related not only to available amino acids but also to enzymes responsible for the reactions—kinases (responsible for phosphate group attachment) and phosphatases (responsible for phosphate group detachment). In the cow placenta, protein kinase-C activity was detected in cytosolic and particular fractions, with specific proteins identified as substrates for this enzyme [19]. The regulation of the activity of these enzymes may have therapeutic meaning.

An important mechanism regulating the availability of selected amino acids of placental proteins is oxidative stress. Since mild oxidative stress is a well-recognized component of both pregnancy and parturition, its increased intensity may directly affect foetal membrane release and contribute to pregnancy-related disturbances in cows and other species. Kankofer analysed products of protein peroxidation in retained and released bovine placenta and found that the contents of bityrosine (product of peroxidative damage to tyrosine) were higher in cases of retained placenta in both parts [20]. This is consistent with our results, where the intensity of phosphorylation differs in molecular weight and total content between samples coming from healthy cows and animals affected by the retention. The reason could be the damage to tyrosine and the lack of available amino acid for the attachment of the phosphate group.

The content of SH groups can reflect the intensity of peroxidative processes of proteins and to some extent the intensity of glutathionylation [21]. The analysis of SH groups in bovine placenta depicted that the concentrations of GSH showed significantly higher values in placentas without the retention of foetal membranes than with the retention. The opposite relationship was noticed for GSSG levels, which were significantly higher in cows suffering from the retention of foetal membranes than healthy animals [20]. These data are in line with current results, indicating that SH groups are not available for binding glutathione in samples collected from cows with the retention of foetal membranes. Interestingly, human plasma fibronectin (one of the key ECM proteins) has recently been shown to be sensitive to glutathionylation, which may alter the mechanical properties of its fibres and cell behaviour through integrin-mediated signalling [22]. While S-glutathionylation of Na^+^/K^+^ pump and its inhibition might be involved in the mechanism of preeclampsia in women [23]. Therefore, there is an issue worth considering whether oxidative stress-driven glutathionylation may affect ECM structure and contribute to impaired foetal membrane release.

Glycosylation patterns of placental proteins, earlier examined by our group, also vary with gestational age and differ between retained and released placentas in cows [24]. The activity of placental glycosidases, which modify glycoproteins involved in cell adhesion, changes throughout pregnancy and parturition, potentially affecting foetal membrane release [25]. Pregnancy-associated glycoproteins, produced by trophoblast cells, show differential expression in cases of (RFM), suggesting a regulatory role in placental detachment [26].

## 4. Materials and Methods

### 4.1. Biological Material

Placental samples were obtained from pregnant Holstein-Friesian dairy cows at a local abattoir immediately postmortem. Dissection was carried out under sterile conditions directly at the gravid uterine horn to ensure sampling of placentomes from anatomically equivalent sites, thereby reducing biological variability. Collected tissues represented the early to mid-gestation period, 2nd to 7th months of pregnancy (*n* = 4 per month). Samples from selected months of pregnancy were included, as these were the only stages available from cows slaughtered at the abattoir. Gestational age was estimated based on foetal crown-rump length [27], measured from the vertex of the skull to the base of the tail. Since the samples were collected postmortem from animals slaughtered in accordance with approved governmental regulations, no ethics approval was necessary. Additionally, placental tissues were obtained from clinically healthy cows undergoing routine elective cesarean section at term. These samples were further (retrospectively) divided into two groups: not-retained foetal membranes (NRFM, *n* = 6) and retained foetal membranes (RFM, *n* = 6). Foetal membranes were considered retained if they were not expelled within 8–12 h after delivery.

This article does not contain any studies with human participants. All institutional and national guidelines for the care and use of animals were followed (EU Directive 2010/63/EU for animal experiments). Ethical review and approval were waived for this study, as experiments conducted on tissues obtained postmortem from slaughterhouses, as well as the collection of biological material during routine veterinary procedures, do not require approval from an ethical committee (national Act of 15 January 2015 on the Protection of Animals Used for Scientific or Educational Objectives).

### 4.2. Preparation of Homogenates

Placentome samples were manually separated into maternal and foetal parts with great care to avoid cross-contamination and analysed separately. Tissue fragments (approx. 2.2–3.1 g) were homogenized individually on ice using mechanical homogenization (Ultra-Turrax, Ikawerk, Janke & Kunkel, Staufen, Germany) in 2 mL of PBS (0.05 M, pH = 7.2) supplemented with Triton X-100 at a concentration of 1% (Sigma-Aldrich, Poznań, Poland) and a protease inhibitor cocktail (Halt™ Protease Inhibitor Cocktail, 87785, Thermo Scientific™, Warszawa, Poland). The homogenates were centrifuged at 6500× *g* for 20 min at 4 °C, and the supernatants were aliquoted in vials and stored frozen (−20 °C) for subsequent Western blotting as well as total phosphoprotein quantitative determinations. Protein concentration was determined using a colorimetric method (Liquid Cor-Total Protein 60, 2-236, Cormay, Warszawa, Poland).

### 4.3. Phosphoprotein Quantification

The extent of protein phosphorylation was determined in protein samples using Phosphoprotein Phosphate Estimation Assay Kit (23270, Thermo Scientific, Rockford, IL, USA) according to the manufacturer’s instructions. Each sample was analyzed in duplicate. Absorbance at 630 nm was measured spectrophotometrically using a microplate reader (Labsystem Multiskan RC, Warszawa, Poland). Data analysis, including standard curve generation, was carried out with Genesis software (Genesis Lite Version 3.03, Life Sciences, Saint Petersburg, FL, USA). Results were expressed as µg total phosphoprotein per g of total protein in the sample.

### 4.4. Preparation of Homogenates Protein Preparation and Western Blot Analysis

An equal amount of protein (20 µg per line) was separated using 12.5% SDS-PAGE. The separated proteins were then transferred onto PVDF membranes (Invitrogen, pb9320, Carlsbad, CA, USA) using the Trans-Blot^®^ Turbo™ Transfer System (25 V, 30 min, Bio-Rad, Warszawa, Poland), following the manufacturer’s instructions. After transfer, the membranes were blocked for 1 h in the animal-free blocker (SP-5030, Vector Laboratories, local distributor Biokom, Janki, Poland). Subsequently, the membranes were incubated overnight at 4 °C with antibodies against individual protein modifications; details are in Table 1. Following this, the membranes were incubated with AP-conjugated anti-mouse antibodies (rabbit anti-mouse IgG (H + L), HRP, ThermoScientific, 61-6520, Warszawa, Poland) for 2 h at room temperature. For visualisation, 1-Step™ Ultra TMB-Blotting Solution (ThermoScientific, 37574, Warszawa, Poland) was used. Staining for β-actin (ThermoScientific, PA1-183, Warszawa, Poland) was used as an internal control for WB. For transfer control, gels were stained with CBB (Roth, Roti^®^-Blue, A152.1, Zielona Góra, Poland). The signal obtained was analysed using ImageJ version 1.54p 17 software and normalised to the total protein in the same sample to account for variations in protein loading.

### 4.5. Statistical Analysis

Statistical analysis covered the comparison of phosphoprotein kit results as well as the intensity of staining obtained during WB analysis between examined periods of pregnancy as well as between retained and released placental samples. Statistical analysis was carried out using Statistica software (version 13.0, StatSoft, Poland; TIBCO Software Inc., Palo Alto, CA, USA). The normality of data distribution was verified using the Shapiro–Wilk test [28]. Homogeneity of variances was assessed with Levene’s test [29]. Depending on the distribution and variance characteristics, group differences were evaluated using non-parametric tests, including the Kruskal–Wallis test [30], the Mann–Whitney U test [31]. No adjustments for multiple comparisons were applied, as the comparisons were predefined in accordance with the experimental design [32]. Significance was declared if *p* < 0.05. Data are presented as medians with interquartile ranges. Spearman’s rank correlation was applied to evaluate the association between gestational age and concentrations of phosphorylated proteins throughout pregnancy. A 95% confidence intervals (95% CI) for the correlation coefficients (Spearman’s ⍴) were estimated using the Fisher z-transformation. Maternal and foetal placental compartments were compared separately. Pregnancy and parturient samples were analyzed separately.

## 5. Conclusions

The placenta has its own metabolic pathway during pregnancy, and any regulation of its pathways may indicate an appropriate pregnancy and parturition course. Both phosphorylation and glutathionylation form an interconnected network regulating placental function. Any alterations in post-translational modifications in placental tissues may be responsible for pregnancy complications. Consequently, further research into glutathionylation, phosphorylation and other PTMs, including the identification of specific target proteins, may provide new insights into bovine pregnancy physiology and related pathologies such as foetal membrane retention. Moreover, the studies on the regulation of enzymes responsible for PTMs may serve for therapeutic aspects in different disorders.

## Figures and Tables

**Table 1:** List of the antibodies used for Western blot analysis.

Antibody	Company, Catalog Number	Host; Reactivity	Dilution
Phosphoserine Monoclonal Antibody (3C171)	ThermoFisher Scientific, MA1-91608	Mouse monoclonal/IgG1; chemical	1:2000
Phosphothreonine Monoclonal Antibody (PT-5H5)	ThermoFisher Scientific, 13-9200	Mouse monoclonal/IgG2a, kappa; chemical	1:1000
Phospho-Tyrosine Monoclonal Antibody (pY20)	ThermoFisher Scientific, 14-5001-82	Mouse monoclonal/IgG2b, kappa; chemical	1:2000
Glutathione Monoclonal Antibody (D8)	ThermoFisher Scientific, MA1-7620	Mouse monoclonal/IgG2a; chemical	1:2000
beta Actin Monoclonal Antibody (BA3R)	ThermoFisher Scientific, MA5-15739-HRP	Mouse monoclonal/IgG2b; dog, chicken, human, mouse, non-human primate, rabbit, rat	1:5000

## Data Availability

The original contributions presented in this study are included in the article. Further inquiries can be directed to the corresponding author.

## Supplementary Materials

The supporting information can be downloaded at: https://www.mdpi.com/article/10.3390/ijms262210984/s1. Reference [33] is cited in the supplementary materials.

## Abbreviations

The following abbreviations are used in this manuscript:

PTM	post-translational modification
GS-ylation	protein glutathionylation
ECM	extracellular matrix
RFM	retention of foetal membranes/retained foetal membranes
NRFM	not-retained foetal membranes
CI	confidence interval
