# Supplementary material for: Selected Post-Translational Modifications—Phosphorylation and Glutathionylation—As Factors Involved in the Regulation During the Pregnancy Course and Foetal Membrane Release in Cows
Source: Int J Mol Sci. 2025 Nov 13;26(22):10984. doi: 10.3390/ijms262210984 (PMC12652969; doi:10.3390/ijms262210984)
Supplement: Supplementary file 1 [file ijms-26-10984-s001.zip › ijms-3910593-supplementary.pdf]

Supplementary Materials

Figures of SDS-PAGE and WB analyses, for example, samples of bovine placenta, are presented below. The SDS-PAGE were stained with Coomassie brilliant blue, and Western blot analysis staining with specified antibody (see Section 4.4 in Materials and Methods and Table 1).

Analysis of the loading control revealed the presence of 42 kDa β-actin. Cleavage of β-actin by enzymes like caspase-3 can result in smaller fragments, such as a ~32 kDa fragment. This is a common hallmark of apoptosis; degradation produces 38–41 kDa fragments on a Western blot (Zhouheng, Y.; Bradley, P.A.; Frank, R.S.; Xinhua, Z. Cleaved β-Actin May Contribute to DNA Fragmentation Following Very Brief Focal Cerebral Ischemia. *J. Neuropathol. Exp. Neurol.* **2018**, *77*, 260–265, doi.org/10.1093/jnen/nly003).

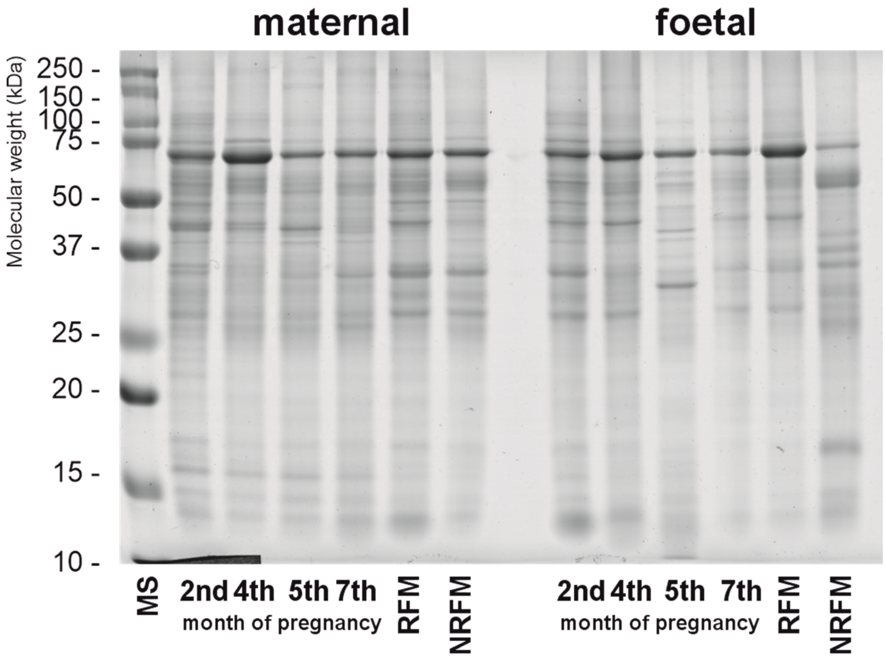

Figure S1. Protein SDS-PAGE analysis of the bovine placenta proteins. RFM—retained foetal membranes, NRFM—not-retained foetal membranes, MS—molecular weight standard (BioRad, #161-0374).

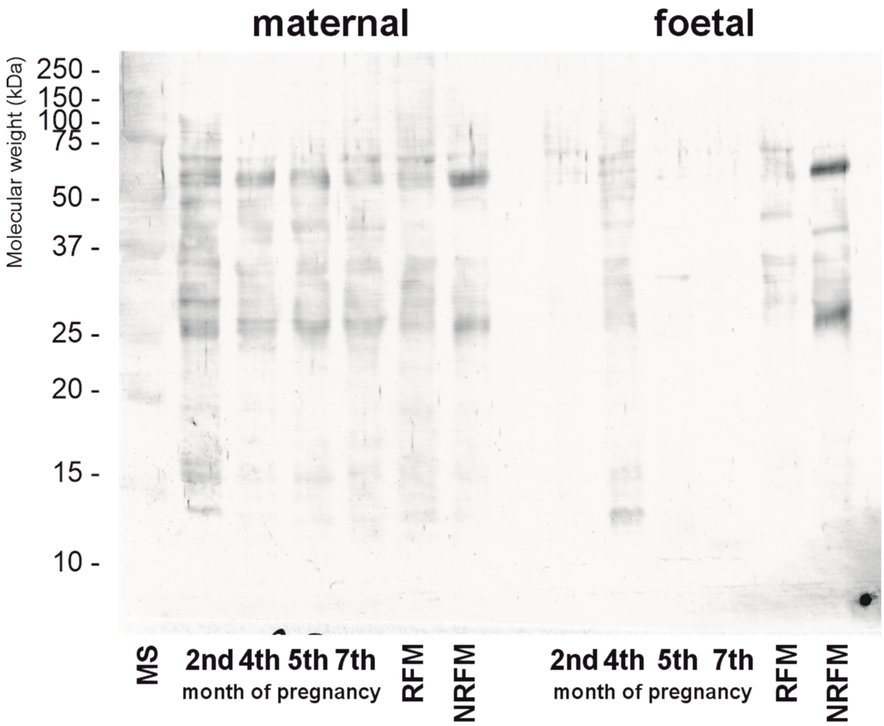

Figure S2. Western blot analysis of the proteins with phosphorylated tyrosine present in SDS-PAGE analysis of the bovine placenta proteins. RFM—retained foetal membranes, NRFM—not-retained foetal membranes, MS—molecular weight standard (BioRad, #161-0374).

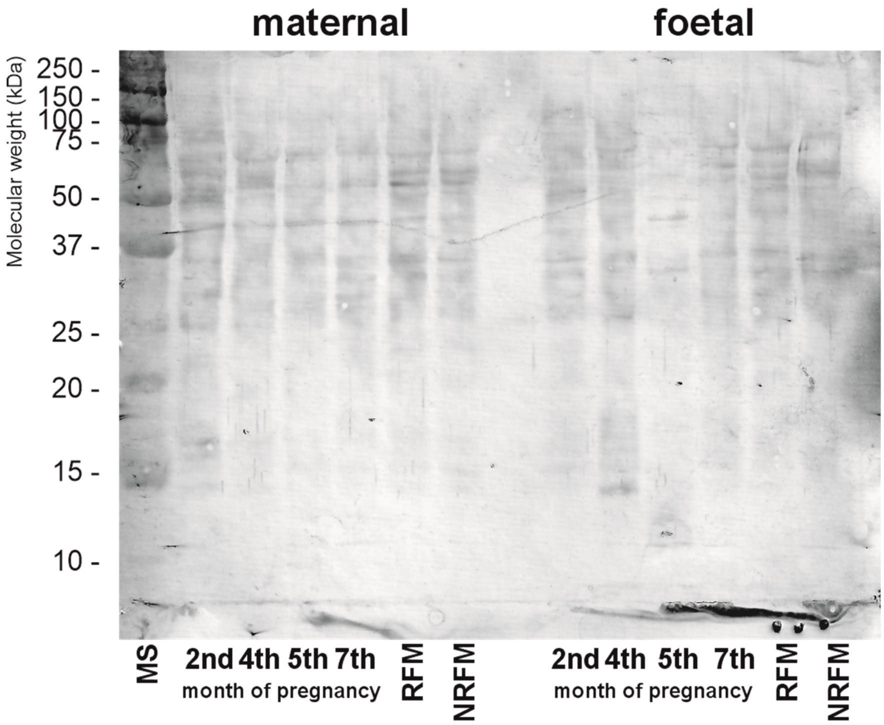

Figure S3. Western blot analysis of the proteins with phosphorylated serine present in SDS-PAGE analysis of the bovine placenta proteins. RFM—retained foetal membranes, NRFM—not-retained foetal membranes, MS—molecular weight standard (BioRad, #161-0374).

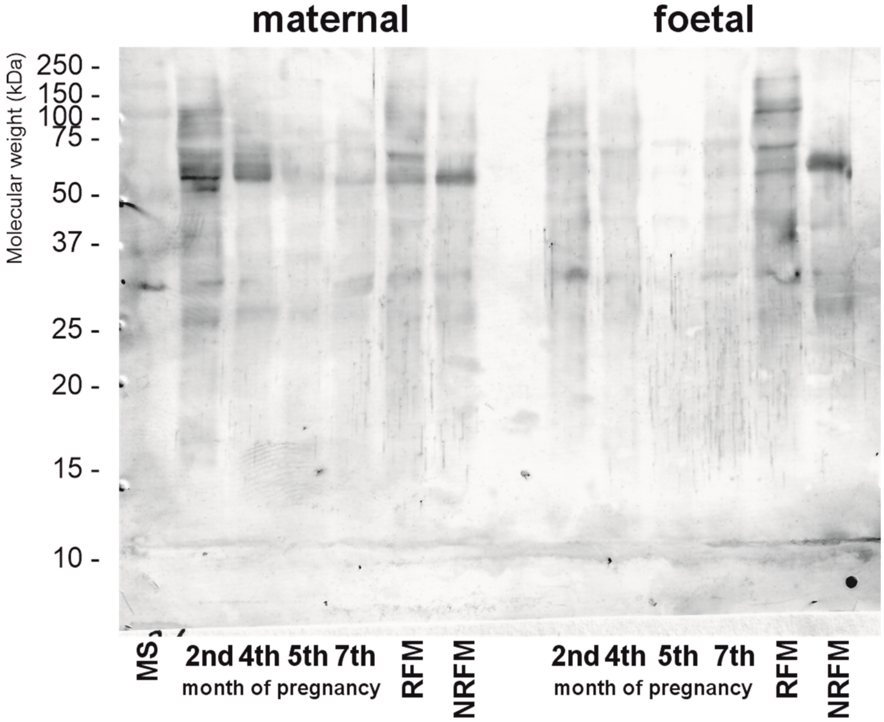

Figure S4. Western blot analysis of the proteins with phosphorylated threonine present in SDS-PAGE analysis of the bovine placenta proteins. RFM—retained foetal membranes, NRFM—not-retained foetal membranes, MS—molecular weight standard (BioRad, #161-0374).

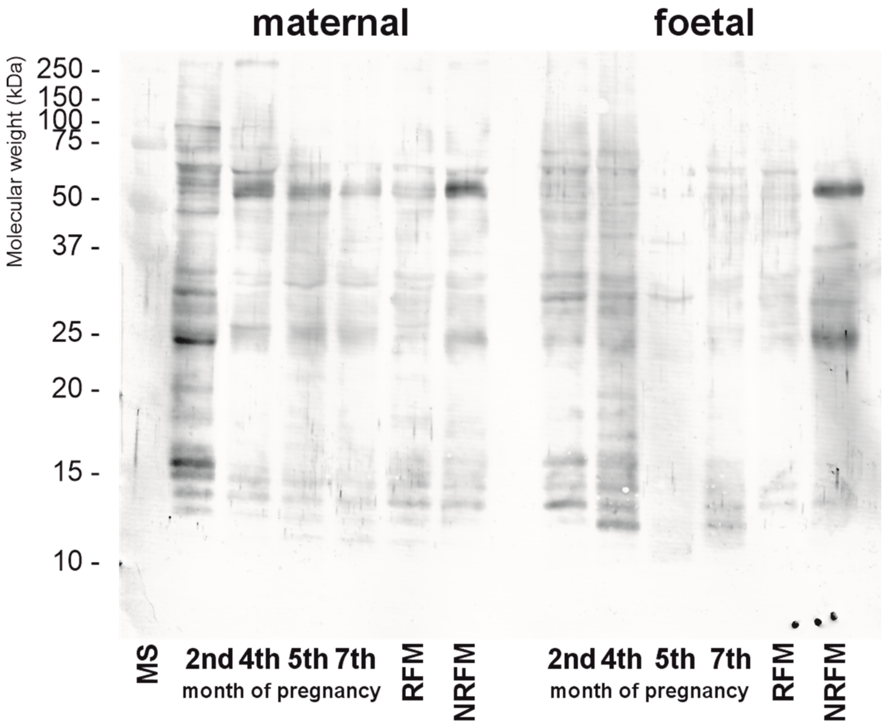

Figure S5. Western blot analysis of the proteins with GSH present in SDS-PAGE analysis of the bovine placenta proteins. RFM—retained foetal membranes, NRFM—not-retained foetal membranes, MS—molecular weight standard (BioRad, #161-0374).

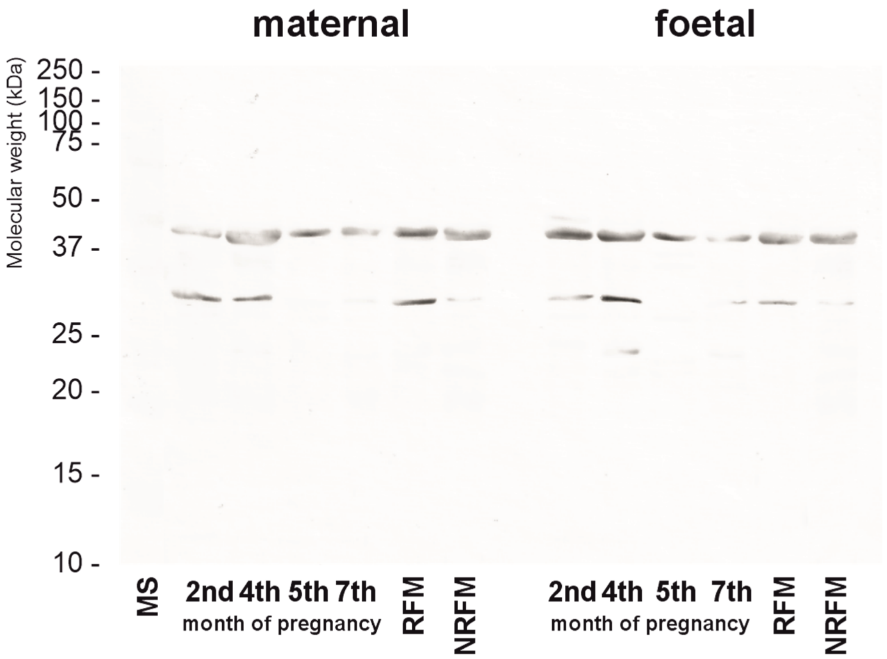

Figure S6. Western blot loading control β-actin in SDS-PAGE analysis of the bovine placenta proteins. RFM—retained foetal membranes, NRFM—not-retained foetal membranes, MS—molecular weight standard (BioRad, #161-0374).
